# Progressive multifocal leukoencephalopathy in non-HIV patient

**DOI:** 10.1590/S1980-5764-2016DN1002015

**Published:** 2016

**Authors:** Diego Rosseman Vieira, Diogo Zanella, Eurípedes Barsanulfo de Paula Avelino, Renato Batista Soares Moura, Gislaine Cristina Lopes Machado Porto, Liao Shin Yu, Rubens Chojniak

**Keywords:** progressive multifocal leukoencephalopathy, non-HIV, lymphoma, JC virus

**Figure 1:**
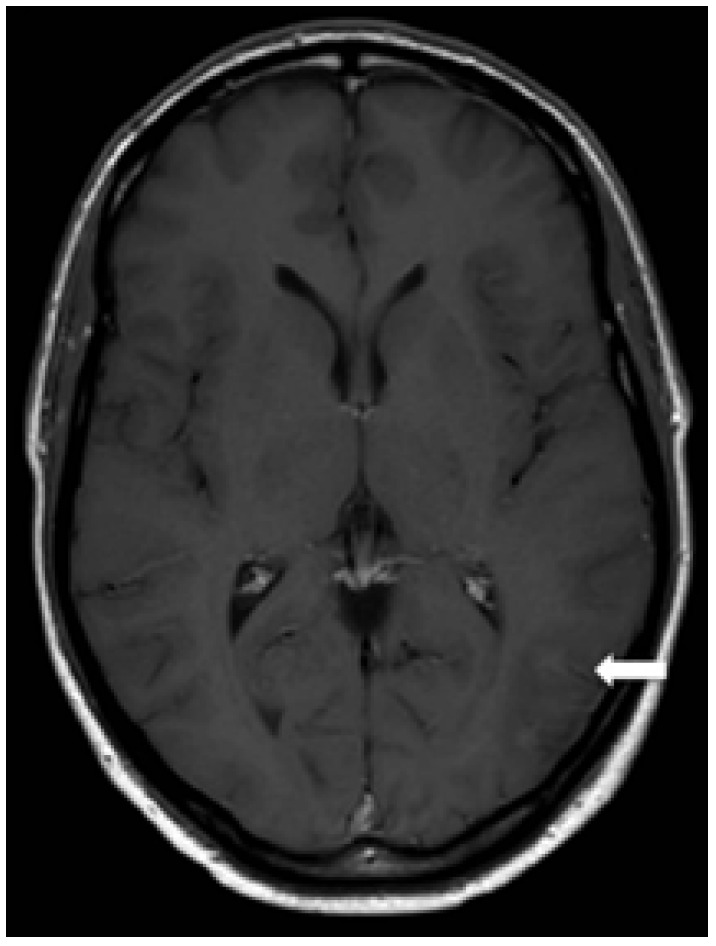
T1-weighted image post-contrast reveals cortical-subcortical hypointensity in left temporal lobe, without enhancement (white arrow).

**Figure 2:**
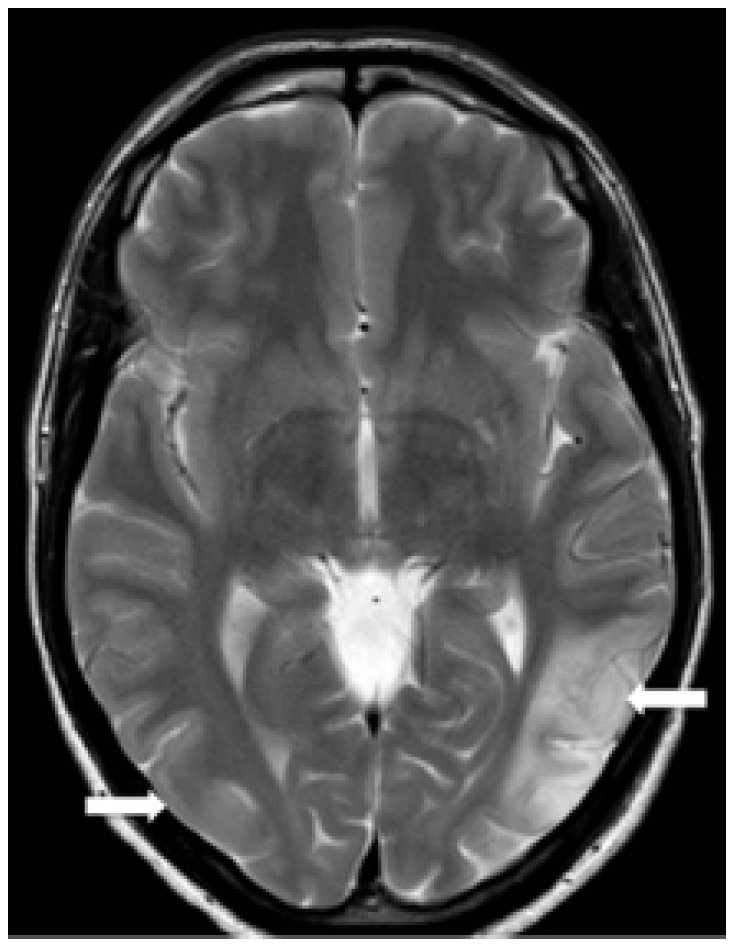
T2-weighted image reveals asymmetrical areas of hyperintensity, more marked in the left temporal region (white arrows).

**Figure 3:**
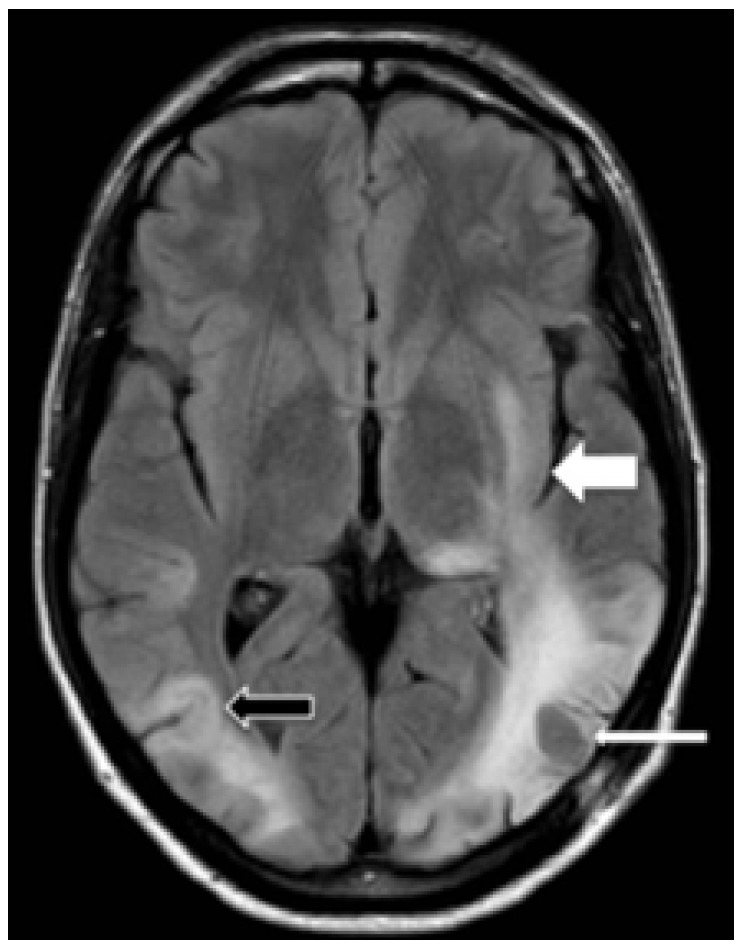
FLAIR image reveals multifocal and asymmetric progression of PML, with involvement of the left temporal-occipital lobes (white arrow) and of the subcortical region of the right temporal lobe (U-fibers) (black arrow). Previous biopsy area (thin white arrow).

We report the case of a 42-year-old man with an indolent non-Hodgkin lymphoma (stage IV) diagnosed 15 months earlier. Since diagnosis, he had remained asymptomatic and in clinical follow-up, under no specific therapy. During a follow-up visit, he reported speech and visual difficulties. Cerebrospinal fluid analysis was normal and serologic tests for herpes virus, syphilis, toxoplasmosis and HIV were all negative. MRI disclosed multifocal asymmetric areas of hypointensity on T1 (Figure 1) and hyperintensity on T2/FLAIR (Figure 2), affecting both temporal and parietal lobes, with no mass effect, *diffusion restriction* or contrast enhancement. Stereotactic biopsy was performed and the anatomopathological examination revealed areas of intense demyelination with presence of large oligodendrocytes Immunohistochemistry staining on brain biopsy demonstrated JC virus DNA, characterizing progressive multifocal leukoencephalopathy (PML). Despite the support therapy, the patient evolved with clinical and radiological deterioration (Figure 3).

PML is a severe demyelinating disease of the central nervous system (CNS). It is caused by the reactivation of the JC virus (genus: Polyomavirus), which infects nearly 80% of the human population prior to adulthood. The virus remains latent mainly in the kidneys and lymphoid organs and is reactivated and spreads to the brain, almost exclusively in the setting of advanced immunosuppression. Once reactivated in the CNS, the virus infects and destroys oligodendrocytes, which are responsible for the formation and maintenance of myelin sheaths.^1^ ^-^ ^3^

PML is frequently associated with HIV/AIDS, presenting in up to 80% of cases. However, other causes of immunosuppression such as organ transplantation, chemotherapy, immunotherapies with monoclonal antibodies, autoimmune diseases and lymphoma, may also be associated with PML. *The clinical picture of* PML varies according to the *patterns of demyelination.* Patients may experience *changes* in *cognition, language*, behavior and *personality*. Memory loss, visual deficits such as hemianopsia and cortical blindness, sensory-motor alterations, and generalized neurological decline, may also occur. The presence of seizures, vertigo, headache and aphasia is less frequent. Cerebellar symptoms may appear in the case of lesion in the posterior fossa. Rarely, there is involvement of the spinal cord.^1^ ^,^ ^3^ The employment of PCR technique for the detection of virus DNA in cerebrospinal fluid, associated with imaging exams, has become an alternative to anatomopathological study, which confirms the diagnosis.^1^

In general, demyelination occurs in a multifocal and asymmetric manner. The lesions typically start at the cortical junction between the gray and white matter, with concentric dissemination. They can be variable in size and may coalesce. There is a predilection for the parieto-occipital lobes.^1^ ^,^ ^3^ MRI is the most sensitive *imaging modality*, particularly in sequences with long *repetition time*. The lesion appears as hypersignal areas on T2 and FLAIR in the subcortical and periventricular white matter. The areas are small in the beginning, increasing progressively, with a tendency for confluence. It is commonly multifocal and has an asymmetric pattern *of demyelination*, uni- or bilaterally, without mass effect, and invariably without contrast enhancement. The subcortical lesions tend to involve U-fibers. Any lobe of the brain may be affected, but there is preference for the frontal and parieto-occipital regions. There may be involvement of deep gray structures, by affection of myelin fibers that pass through *basal ganglia*. In about one third of cases, there is involvement of the posterior fossa and compromise may be limited to this area in 10% of cases.^1^ ^,^ ^5^ ^,^ ^6^

The differential diagnosis of PML includes other CNS diseases that can occur in immunocompromised patients, such as toxoplasmosis and primary lymphoma of the CNS. PML can be differentiated from these diseases by its slower temporal evolution, fewer systemic manifestations, absence of fever, preservation of conscience and lack of mass effect, perilesional edema or contrast enhancement on imaging tests. Herpes virus encephalitis should be considered in the initial phase, but white matter involvement is frequently temporal, bilateral and asymmetrical, with greater contrast enhancement than that found in PML.^5^ ^,^ ^6^ Another important differential is HIV-associated demyelination. It may be difficult to distinguish between the demyelinating lesions caused by PML or by HIV, in patients with AIDS. PML lesions are more often multifocal and asymmetric, with greater predilection for the subcortical white matter and are associated with more pronounced signal change on T1-weighted images, while in HIV-associated demyelination, lesions are often isointense on T1, and may not be visible. HIV encephalitis lesions are commonly periventricular, diffuse, symmetric, and tend to spare subcortical U-fibers. Clinically, HIV-associated demyelination presents as global cognitive disorder and dementia, while in PML motor, sensory or cognitive focal deficits predominate.^1^ ^,^ ^5^ ^,^ ^6^

The prognosis for PML is reserved and there is no specific therapy. Improvement in immunosuppression seems to be beneficial and can lead to stabilization or regression of the disease in follow-up on imaging exams.^1^
